# A genome-wide study of PDZ-domain interactions in *C. elegans *reveals a high frequency of non-canonical binding

**DOI:** 10.1186/1471-2164-11-671

**Published:** 2010-11-26

**Authors:** Nicolas Lenfant, Jolanta Polanowska, Sophie Bamps, Shizue Omi, Jean-Paul Borg, Jérôme Reboul

**Affiliations:** 1 Inserm, U891, CRCM, Marseille, F-13009, France; Institut Paoli-Calmettes, Marseille, F-13009, France; Univ Méditerranée, F-13007, Marseille, France; 2 Institute of Integrative and Comparative Biology, Faculty of Biological Sciences, The University of Leeds, Leeds, LS2 9JT, UK; 3 CIML, Parc Scientifique et Technologique de Luminy Case 906 - 13288 Marseille - Cedex 9, France

## Abstract

**Background:**

Proteins may evolve through the recruitment and modification of discrete domains, and in many cases, protein action can be dissected at the domain level. PDZ domains are found in many important structural and signaling complexes, and are generally thought to interact with their protein partners through a C-terminal consensus sequence. We undertook a comprehensive search for protein partners of all individual PDZ domains in *C. elegans *to characterize their function and mode of interaction.

**Results:**

Coupling high-throughput yeast two-hybrid screens with extensive validation by co-affinity purification, we defined a domain-orientated interactome map. This integrates PDZ domain proteins in numerous cell-signaling pathways and shows that PDZ domain proteins are implicated in an unexpectedly wide range of cellular processes. Importantly, we uncovered a high frequency of non-canonical interactions, not involving the C-terminus of the protein partner, which were directly confirmed in most cases. We completed our study with the generation of a yeast array representing the entire set of PDZ domains from *C. elegans *and provide a proof-of-principle for its application to the discovery of PDZ domain targets for any protein or peptide of interest.

**Conclusions:**

We provide an extensive domain-centered dataset, together with a clone resource, that will help future functional study of PDZ domains. Through this unbiased approach, we revealed frequent non-canonical interactions between PDZ domains and their protein partners that will require a re-evaluation of this domain's molecular function.

[The protein interactions from this publication have been submitted to the IMEx (http://www.imexconsortium.org) consortium through IntAct (PMID: 19850723) and assigned the identifier IM-14654]

## Background

Because of its biological importance, the PDZ (PSD-95, Discs-large, ZO-1) domain has been intensively studied at the structural and functional level. Proteins containing PDZ domains frequently serve as molecular scaffolds, which assemble signaling complexes needed for efficient and specific signal transduction at defined sub-cellular sites, such as at polarized epithelial cell junctions, or synapses in neurons [1-3]. Early work indicated a preferential interaction between PDZ domains and the C-terminal amino acids of target proteins [4]. In some cases, removal of the 3 C-terminal residues of the partner protein abrogates interaction with the PDZ domain [5]. Much subsequent effort has been put into bioinformatic studies and small- and large-scale screens to refine the exact sequence of this presumed C-terminal motif [5-10], leading to several consensus sequences, with different degrees of refinement (e.g. Additional file 1; [1,10-12]). Individual proteins can contain multiple PDZ domains. For example, the human multiple PDZ domain protein (MPDZ) has 13. When their interactions with other proteins have been dissected, the different PDZ domains of a single protein often have been found to have distinct binding partners (see for example the Uniprot entry for MPDZ [13]). PDZ domain proteins have also been used in the context of large-scale searches for protein partners. For example, global interactome studies with *C. elegans *proteins assayed the interactions of 25 of the nematode's 62 PDZ domain proteins. Although these 25 proteins were found to be involved in 218 interactions, whether the different PDZ domains played a direct role was not addressed [14,15]. No comprehensive, proteome-wide screen using all PDZ domains, however, has been reported for any organism.

Here, we describe the characterization and cloning of every single one of the 93 PDZ domains from *C. elegans*. We generated a versatile resource, with each domain in the Gateway system, allowing facile transfer to different expression systems. As an example, we made a yeast array of the 93 PDZ domains and provide a proof-of-principle for its application to the discovery of PDZ domain targets for any protein or peptide of interest. In addition, from a separate yeast two-hybrid (Y2H) screen, we identified more than 650 potential partners for these domains. A large number of these interactions were independently validated using a co-immunoprecipitation approach. An analysis of these interactors implicates PDZ domains in a broad range of cellular functions. Unexpectedly, many of the interactions did not involve a C-terminal consensus sequence, suggesting that PDZ domains frequently bind their partners in a hitherto uncharacterized mode.

## Results

### An interactome map for PDZ domains

We chose to define the interaction partners of all the PDZ-domain proteins in *C. elegans*. Through an exhaustive cross-database search, we identified a total of 93 PDZ domains in 62 distinct proteins, not counting isoforms sharing domains (see Additional file 2: Supplemental Table S1). Among these PDZ-domain containing proteins, only 44% were associated with any gene ontology annotation based on experimental data ([16]; Additional file 2: Supplemental Table S2). The DNA for all 93 domains was amplified and cloned. The insert for each clone was sequenced-verified, and this comprehensive clonal collection, in the Gateway entry vector allowing rapid transfer into multiple other vectors [17,18], is available as a community resource upon request. The inserts were all transferred into a DB-vector and used in high-stringency Y2H screens against the non-normalized cDNA library AD-wrmcDNA [18]. We pulled out 447 interactions involving 317 interacting proteins and 75 individual PDZ domains. 6 PDZ domains were auto-activators and therefore not included in the screen, thus 81% of the PDZ domains gave at least 1 interaction, with a mean of 6 interacting proteins (see Additional file 3: Supplemental Tables S3, S4 and S5). As expected, there was limited overlap with the results of the previous global *C. elegans *Y2H screens due in part to the incomplete and disparate degrees of coverage (6 shared interactions with Worm Interactome 8 [15]), and the domain-nature of the current screen. We did observe a clear bias towards proteins containing C-terminal class I consensus motifs (as defined in Additional file 1). One striking observation, however, was the high frequency (51%) of interacting proteins that did not possess a classical C-terminal consensus sequence (Table 1; Additional file 3: Supplemental Table S6). This trend was maintained even when interacting proteins that had multiple PDZ-domain partners were counted only once in the analysis (Table 1). This opened the possibility to perform a second screen using the AD-ORFeome library [19], which has the advantage of being highly normalized. In this library, the stop codon of each insert is replaced by the B2 recombination sequence, giving rise to proteins with a constant non-native C-terminal extension. These additional 8 amino acids (PAFLYKVV) do not correspond to the consensus binding sequence for native PDZ-domains. Using this library, we identified a total of 227 interactions involving 178 interacting proteins and 59 PDZ domains (see Additional file 3: Supplemental Table S3). These included 14 in common with the cDNA screen. This degree of overlap (6%) is slightly lower than that reported [14,19] for previous screens against the two libraries (14% and 16%, respectively), possibly reflecting the fact that our PDZ domain screen is biased towards native C-terminus consensus motifs, which are not accessible in the AD-ORFeome library. Within these 59 PDZ domains (63% of the total; mean 3.8 interacting proteins per domain; see Additional file 3: Supplemental Tables S4 and S5), the prevalence of C-terminal consensus sequences in the interacting proteins reflected that seen in the proteome as a whole (Table 1; Additional file 3: Supplemental Table S6). Taken together, these results suggest that some PDZ domains might interact with their target ligands outside the C-terminus much more often than expected.

**Table 1:** Proportions of C-terminal consensus classes in interacting proteins.

	consensus class 1	consensus class 2	consensus class 3	total consensus	total non consensus	
AD-wrmcDNA library	20%	24%	5%	49%	51%	n = 447
AD-ORFeome library	10%	18%	5%	33%	66%	n = 227
*C. elegans *proteome	8%	18%	5%	31%	69%	n = 20186

	**consensus class 1**	**consensus class 2**	**consensus class 3**	**total consensus**	**total non consensus**	

AD-wrmcDNA library	18%	21%	5%	44%	56%	n = 317 nr
AD-ORFeome library	9%	15%	4%	28%	72%	n = 178 nr
*C. elegans *proteome	8%	18%	5%	31%	69%	n = 20186

Proportions of C-terminal consensus classes for interacting proteins identified in AD-wrmcDNA and AD-ORFeome screens compared with proportions of C-terminal consensus classes in the complete *C. elegans *proteome (WS190 [16]) are given. Consensus class 1: [ST]X[FWCYMVILA], Consensus class 2 [YFWCMVILA]X[YFWCMVILA], Consensus class 3 [DE]X[YFWCMVILA] (X: any amino acid). Upper panel: proportions calculated using all PDZ-interacting protein pairs, lower panel: proportions calculated using each interacting protein only once when interacting with multiple PDZ domains (nr stands for non-redundant).

### Characteristics of PDZ domain interacting proteins

**Figure 1:**
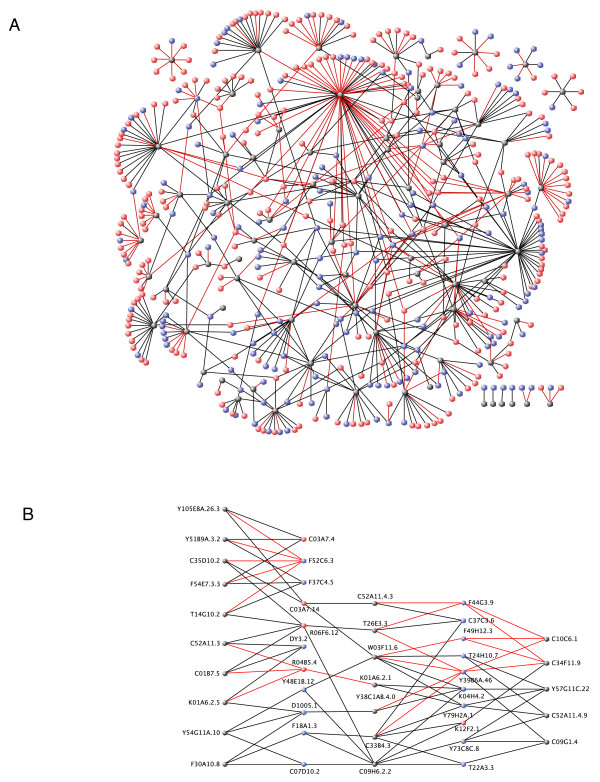
**Yeast two-hybrid interactome maps of PDZ domain interactions**. Grey nodes represent individual PDZ domains, blue nodes represent interacting proteins having a C-terminal consensus sequence (as defined in Additional file 1) and red nodes represent interacting proteins that do not have a C-terminal consensus sequence. Red edges represent interactions identified in the AD-ORFeome yeast two-hybrid screen and black edges represent interactions identified in the AD-wrmcDNA yeast two-hybrid screen. (A) Global representation of the 674 interactions involving 469 proteins and 78 PDZ domains. (B) Promiscuity: representation of interactions involving selected target proteins with multiple PDZ domains. For multiple PDZ domains in the same protein, a ".n" extension numbered from the ATG was added to the ID of the PDZ containing protein. Some interactions have been omitted for the sake of clarity. Graphs were designed using VisANT [53].

**Figure 2:**
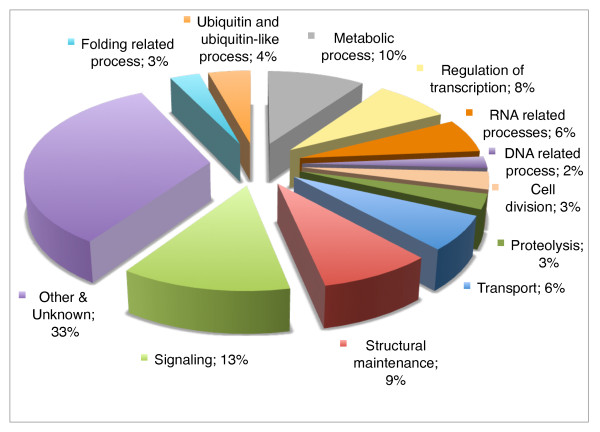
**Classification of interacting proteins according to Biological Processes**. Manual curation of the annotations for each interacting protein when available were retrieved from Wormbase WS190 [16] and used to define the 11 groups of processes shown. PDZ domain proteins appear to be involved in a disparate range of functions.

Pooling the results from the two screens, we obtained 674 interactions involving 469 proteins and 78 PDZ domains (from 55 proteins out of the original 62)(Figure 1A). In many cases, single proteins were found to interact with multiple PDZ domains (Figure 1B), consistent with the known promiscuity of ligand-PDZ domain interactions [20]. Gene Ontologies (GO) [21] analysis are limited by the fact that only 182/469 proteins have attributes inferred from experimental evidence. We therefore opted to extend this analysis with a manual curation of our protein set based principally on Wormbase [16] annotations (Figure 2, Additional file 4: Supplemental Tables S7, S8 and S9). PDZ domains have long been known to be involved in the scaffolding of proteins complexes at the plasma membrane thus contributing to the signaling specificity of many receptors, notably at the synapse [22], or to the establishment and maintenance of epithelial polarity [2,23]. Indeed, just under half of the functionally annotated proteins are involved in signaling (protein kinases, GTPases and phosphatases), structural maintenance or transport. The annotation of the other PDZ interacting proteins reveals a broad range of functions ranging from metabolism, ubiquitination, RNA binding and processing to transcriptional regulation (Figure 2). Interestingly some studies indicate nuclear roles for PDZ domain proteins. For example, the junctional protein ZO-2 directly interacts in the nucleus with the DNA-binding protein scaffold attachment factor-B (SAF-B) [24].

### Frequent use of non-consensus binding confirmed by co-IP

**Figure 3:**
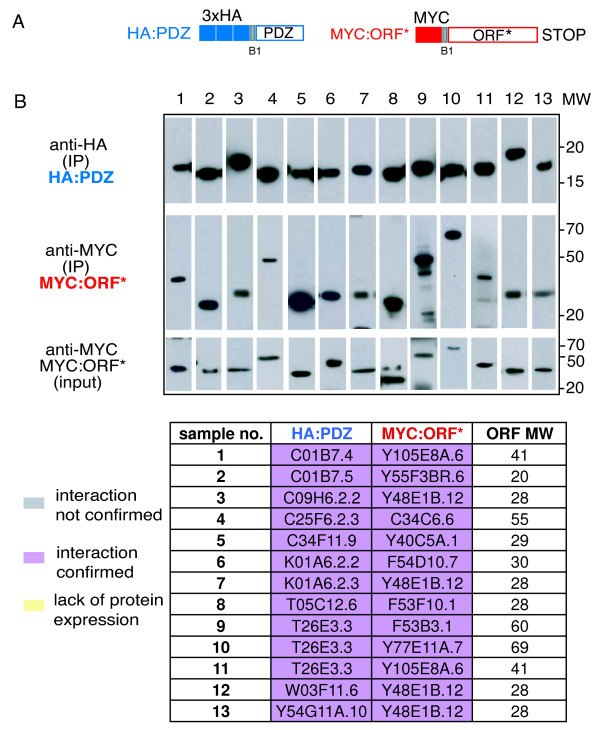
**Co-IP verification of yeast two hybrid interacting pairs identified in AD-wrmcDNA screen**. (A) Schematic representation of PDZ domains carrying N-terminal 3XHA epitope tag and of their full-length interaction partner terminated with a stop codon (ORF*) carrying N-terminal MYC epitope. Each corresponding pair of constructs to be tested was co-expressed in 293T cells and cellular lysates were subjected to precipitation with anti-HA Sepharose. (B) Presence of interacting protein upon precipitation was revealed by western blotting using anti-MYC serum. For each IP performed three panels are presented. Upper panel: IP reaction probed upon resolution on SDS-PAGE and blotting with anti-HA antibody detecting HA-PDZ domain; Middle panel: the same IP reaction probed with anti-MYC serum detecting ORF (MYC:ORF*); lower panel: detection of expression of each ORF by probing total crude cellular extracts (input) with anti-MYC serum. Table summarizes the interaction pairs tested.

**Figure 4:**
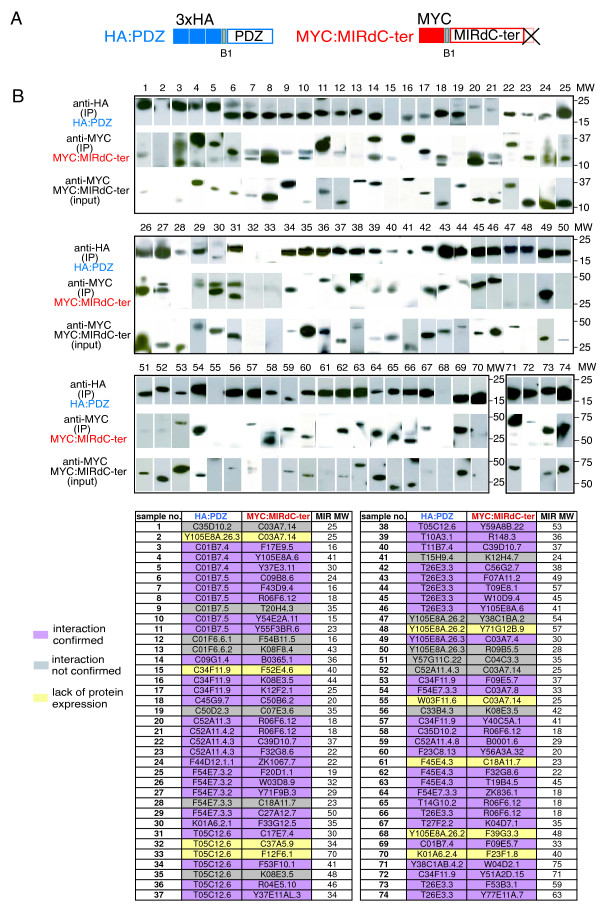
**Verification of internal binding of PDZ-domain and non-consensus C-terminally truncated protein partner by co-immunoprecipitation**. (A) Schematic representation of both tagged proteins: PDZ domains carrying an N-terminal 3XHA epitope tag and C-terminally truncated (dCter) Y2H interacting protein fragment (MIR: experimentally defined minimal interaction region) carrying N-terminal MYC epitope. (B) All pairs were co-expressed in 293T cells and co-IPed using anti-HA sepharose beads. Binding of given protein upon precipitation was revealed by western blotting using anti-MYC serum. For each IP performed three panels are presented. Upper panel: IP reaction probed after resolution on SDS-PAGE and blotting with anti-HA antibody. Middle panel: the same IP reaction probed with anti-MYC serum detecting the truncated protein fragments (MYC:MIRdCter). Lower panel: detection of expression of each truncated protein fragment by probing total crude cellular extracts (input) with anti-MYC serum. The table identifies interaction pairs by their lane number and order in which they are presented in blot panels. The color code summarizes the outcome for each pair (purple: interaction tested positive, grey: no interaction detected and yellow: inconclusive as one or both partners were not expressed). Each MYC:MIRdCter construct used in above co-IP experiment was also subjected to co-transfection and co-immunoprecipitation with empty pDEST-CMV-3xHA vector to serve as a negative control for the binding assay (see Additional file 7).

It is well established that some Y2H interactions do not reflect a physiologically relevant binding events between proteins. Ideally, these interactions need to be validated in vivo. Such tests are fastidious and not compatible with large-scale studies. We therefore sought to assay a subset of our Y2H interactions, using a very distinct experimental system, namely co-affinity immunoprecipitations (co-IP) from human 293T cells. First, we choose 13 different interactions, found in the cDNA screen, involving 11 PDZ domains and 9 interacting proteins. All these interactions were detected by co-IP using constructs encompassing the PDZ interacting full-length proteins, with their native C-terminus (Figure 3, Additional file 5: Supplemental Table S10). We then extended the co-IP test and used B2-tagged constructs, i.e. giving proteins with a non-native and non-consensus C-terminus (see above), to test 38 putative interactions. Of these, we could test 31 interactions (15 from the cDNA screen, 9 from AD-ORFeome screen, and 7 from both. 27 interactions (87%) detected by Y2H were reproduced in these tests, including 12/15 cases where the Y2H interaction had originally been found only in the cDNA screen using constructs with a native C-terminus (see Additional file 6, Additional file 5: Supplemental Table S11). We thus confirmed many interactions between PDZ domains and proteins with a non-native and/or non-consensus C-terminus. To investigate further the possibility that these PDZ domains were interacting with an internal sequence in the partner protein, we returned to a set of high-confidence interactions found in the cDNA screen. For 59 proteins (corresponding to 74 interactions) that did not possess a canonical consensus C-terminal, we cloned derivatives corresponding to the entire protein less the 3 last residues, or when possible the experimentally defined minimal interacting region (MIR), also without the 3 C-terminal residues. This was to ensure that observed interaction did not depend on the native C-terminal residues. Using co-IP, we found that 52/65 interactions (80%) successfully tested could be reproduced in the co-IP system even in the majority of cases when removal of the terminal residues did not create a new consensus binding site (Figure 4, Additional file 7, Additional file 5: Supplemental Tables S12 and S13). We are therefore confident that the dataset that we provide will be a useful source of information to direct studies of PDZ-domain signaling pathways.

### A Y2H array as a tool to probe PDZ domain binding

**Figure 5:**
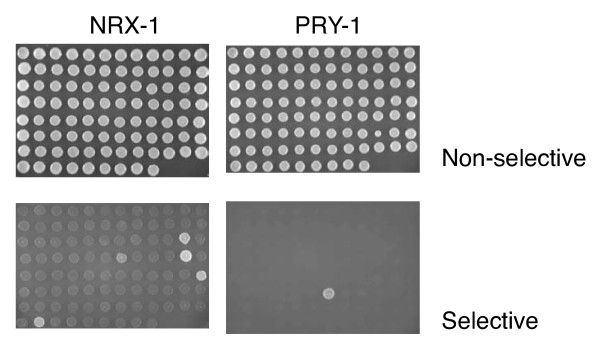
**Y2H array for detecting interactions with PDZ domains**. An array of yeast strains each expressing one of the 93 *C. elegans *PDZ domains, together with a marker permitting growth on medium lacking leucine, and containing a second vector allowing growth on medium lacking tryptophan as well as expressing NRX-1 (left-hand panels) and PRY-1 (right-hand panels), spotted onto solid agar medium. The upper row shows that all strains grow on medium lacking leucine and tryptophan indicating that all strains contain both prey and bait vectors. In the lower row, on fully selective medium, lacking leucine, tryptophan and histidine, growth only occurs when there is an interaction between a PDZ domain and the protein of interest.

Many true interactions protein-protein interactions are missed in Y2H library screens. This high rate of false negatives can be partially alleviated by performing directed Y2H assays [25-27]. We therefore decided to construct a Y2H interaction array that would allow candidate proteins to be screened for their binding capacity to the comprehensive set of PDZ domains. For this, we took a collection of yeast strain each expressing a single PDZ-domain from an Y2H AD-vector, and spotted them in a standard 8 × 12 format on a solid agar support. The individual domains on the array can be probed by introducing into each strain a vector allowing the expression of a protein of interest, using a standard Y2H approach. To test the utility of the resource, we first conducted parallel matings with a yeast strain expressing NRX-1, the *C. elegans *ortholog of vertebrate neurexin, which plays a critical role in synaptic development (Figure 5). Consistent with the results of our Y2H screen that had identified NRX-1 as an interactor of SYD-1, we found SYD-1 as a partner for NRX-1 using the array. SYD-1 is also a regulator of synaptogenesis. We also identified an additional 4 binding partners, including the single PDZ domain containing STN-2, a gamma syntrophin, and MPZ-1 that can be found at synapses. MPZ-1 has 10 PDZ domains and we detected an interaction only with the 9th domain. We also screened the array with LET-23 and identified 4 proteins, including its known partner LIN-7 [28] (data not shown). On the other hand, when we screened the array with PAC-1, we found PAR-6, which has been demonstrated to be its physiological functional partner [29] (data not shown). Similarly, the sole interactor identified for PRY-1, a negative regulator of Wnt signaling, was MIG-5, one of three *C. elegans *Dishevelled homologs that functions in both canonical and non-canonical Wnt signaling pathways (Figure 5). Neither of the other 2 Dishevelled homologs were detected as interacting with PRY-1, consistent with previous studies [30].

## Discussion

We constructed a comprehensive, proteome-wide interaction map for all the PDZ domains from *C. elegans*. Importantly, for a substantial proportion of the interactions, we were able to obtain independent biochemical confirmation. The interactions we characterized covered a broad range of putative biological functions, reflecting the ubiquitous involvement of PDZ-domain proteins in cellular physiology. Although a number of PDZ-domain proteins have been functionally characterized in great details, in very few cases has a role for an individual PDZ-domain been identified. We did find a small group of interactions involving PDZ-domains protein for which there was prior experimental evidence, such as those involving LET-23 and LIN-7, and PAR-3 and PKC-3 [28,31]. Further, we were able to provide a molecular basis for certain previously characterized genetic interactions (e.g. between the polarity gene *par-6 *and the RhoGAP *pac-*1 [29]). There were many additional interactions that could merit directed study, such as that between PTEN/DAF-18 and Dishevelled/DSH-1, two proteins that function respectively in the PTEN/AKT and WNT pathway, and the multipartite interaction between LIN-7 and CSC-1, and LIN-10 with CSC-1 and ICP-1. The LIN-2/LIN-7/LIN-10 complex is known for its role in basolateral targeting of the LET-23 receptor. CSC-1 and ICP-1 are orthologs of Borealin and Incenp two components of the vertebrate chromosomal passenger complex (CPC). We confirmed CSC-1's interactions and the interactions between the two PDZ domains of LIN-10 with ICP-1 using a biochemical approach (JP, unpublished results). This raises the possibility of an unsuspected functional link between these two protein complexes, and is a good example of the hypotheses that can be generated through global analyses.

As a last example, both via our global screen and using the PDZ-domain array, we detected an interaction between MIG-5 and PRY-1. Previous studies had mapped the interaction between PRY-1 and MIG-5 to the N-terminal half of MIG-5 [30], which does contain the protein's single PDZ domain. The C-terminus of PRY-1 (IAAELR) does not contain a consensus PDZ-binding motif. This therefore represents a clear example of a functionally validated protein-protein interaction that we have shown to involve a non-canonical PDZ domain interaction. Indeed, more than half of the interactions did not involve the previously defined PDZ-domain binding C-terminal motifs. By aligning and analyzing our set of PDZ-interacting proteins, we were unable to identify a clear internal motif that could be uniquely responsible for PDZ domain binding. Nevertheless, this global study clearly indicates that non-consensus binding is a much more frequent phenomenon than previously suspected. Extensive future functional studies will be needed to validate all the individual internal PDZ domains interactions described here, but it is important to note that in certain isolated cases, this unconventional mode of binding has been demonstrated [32-42].

It is clear that global Y2H screens only reveal a fraction of potential protein-protein interactions [43]. Among other factors, this is due to cDNA representation in non-normalized libraries. This was one motivation for generating an array that allows direct Y2H assay of any protein or peptide of interest against a complete set of PDZ domains. Coupled with the collection of PDZ domain sequences in the Gateway entry vector, allowing facile transfer to vectors for RNAi, or protein expression, the array, which is available as a community resource, will allow comprehensive functional analyses of all PDZ domains in *C. elegans*.

## Conclusions

By conducting a comprehensive, domain-centered interactome study, we have clearly illustrated at the genome scale the degree of promiscuity and discrimination that governs interactions between individual PDZ domains and their protein partners. This approach also revealed that PDZ domains frequently interact in a non-canonical fashion. This broadens our understanding of PDZ domains and should guide future functional studies.

## Methods

### PDZ domain identifications in *C. elegans *proteome

Domain boundaries where obtained by cross searching Wormbase WS150 [44] and SMART version 4.0 (genomic mode) (http://smart.embl-heidelberg.de) [45]. Each domain was extended on each side with a 10 amino acid tail from the original protein to ensure the integrity of the structure of the domain. In some cases size of these tails had to be slightly modified according to the position of the PDZ in protein (extreme end or start) or to ensure a correct amplification.

### PDZ domain cloning

Primers, containing Gateway B1 and B2 recombination tails, were designed using the OSP program as described [46,47] including a stop codon before the B2 tail (see Additional file 2: Supplemental Table S1). DNA fragments encoding each PDZ domain where amplified by polymerase chain reaction (Platinum HIFI polymerase, Invitrogen) and cloned into pDONR201 Entry vector using the Gateway recombinational cloning system as described [17,18]. PDZ Entry clones were sequence verified using P201DONRF primer 5'-TCGCGTTAACGCTAGCATGGATCTC and then used in a Gateway LR recombination reaction to transfer the DNA coding for the PDZ domain into the yeast expression vector pPC97-Dest as described [18].

### Transformation of pDB-ORFs into yeast cells and removal of auto-activators

DB-ORF plasmids were transformed into yeast strain MaV203 using standard transformation protocols [48]. Auto-activators were identified by testing the activation of *GAL1::HIS3 *on minimal medium lacking leucine and histidine but containing 20 mM 3-amino-1,2,4-triazole (3-AT) in the absence of any AD-containing vector.

### Identification of interacting protein pairs

Bait strains containing a single pDB-PDZ were individually transformed with the *C. elegans *AD-wrmcDNA and AD-ORFeome1.0 libraries [19] as described [48]. A minimum of 1 × 10^6 ^colonies were screened for each bait strain tested with the AD-wrmcDNA library and a minimum of 1.5 × 10^5 ^colonies were screened for each bait strain tested with the AD-ORFeome library. After 4 to 5 days at 30°C, single 3-AT resistant colonies were picked on synthetic complete medium lacking leucine, tryptophan, and histidine and containing 20 mM 3-AT (SC, Leu-, Trp-, His-, 20 mM 3-AT) and then rearrayed on fresh SC, Leu-, Trp-, His-, 20 mM 3-AT plates.

### Phenotypic assays

Colonies able to grow on SC, Leu-, Trp-, His-, 20 mM 3-AT plates were tested for expression of three Y2H reporter genes *(GAL1::HIS3*, *GAL1::lacZ*, and *SPAL10::URA3*, as described [48].

### ORF insert sequencing

To prepare DNA for PCR, yeast colonies were re-suspended in 15 μl lysis buffer (50 units zymolase in 0.1 M Na-Phosphate buffer pH 7.4) using toothpicks, and lysed by incubating for 10 min. at 37°C and 10 min. at 95°C. For each PCR, 0.3 μl of lysis mix was used. AD inserts were amplified using primers 5'-CGCGTTTGGAATCACTACAGGG and 5'-GGAGACTTGACCAAACCTCTGGCG (AD and TERM respectively). DB inserts were amplified using primers 5'-GGCTTCAGTGGAGACTGATATGCCTC (DB) and TERM. PCR products were sequenced using the AD or DB primers.

### Sequence trace analysis

Colonies showing an activation of at least two of the three Y2H reporter genes were PCR amplified, as described above. PCR products showing a single band on ethidium bromide gel were sent for sequencing. The quality of the sequence obtained was determined as described [14] by moving a sliding window of 10 base pairs along the sequence to define the portion that has an average PHRED score of 20 or higher [49,50]. Sequences for which less than 15% of their length met this criterion were discarded. A nucleotide BLAST [51] search was performed against Wormpep150 [44] to determine the identity of the clone. Finally, the reading frame was obtained by local alignment of the 3' end of the Gal4 AD encoding sequence with the 5' end of the prey encoding sequence. A translation according to this reading frame was used to perform a protein BLAST search against Wormpep150. If the nucleotide and protein BLAST agreed, the prey encoding sequence was considered "In Frame", otherwise it was designated as "Out of Frame" and discarded.

### Retesting

Gap repair was used to retest all Y2H interactions as described [48]. When an interaction failed to be re-confirmed it was discarded from the dataset.

### Construction and screening of a comprehensive PDZ domain Y2H array

All PDZ domains were transferred into AD vector (pACT2) by Gateway recombinational cloning and transfected into the haploid Y187 yeast strain (*MAT*α, *ura3-52*, *his3-200*, *ade2-101*, *leu2-3, 112*, *gal4*Δ, *met-*, *gal80*Δ, *MEL1*, *URA3*::*GAL1UAS *-*GAL1TATA*-*lacZ)*. Individual ORFs of proteins of interest were cloned into DB vector (pGBT9) by Gateway recombinational cloning and the resulting constructs transformed into haploid AH109 yeast strain *(MATa, trp1-901, leu2-3, 112, ura3-52, his3-200, gal4*Δ, *gal80*Δ, *LYS2::GAL1UAS-GAL1TATA-HIS3, GAL2UAS-GAL2TATA-ADE2, URA3::MEL1UAS-MEL1TATA-lacZ, MEL1)*. Interactions between each PDZ and a given ORF was tested through mating of the two yeast strains. Phenotypic testing evaluated growth of diploid cells on selective medium (Leu-, Trp-, His-, 2 mM 3-AT), which is dependent in part upon the expression of the GAL1::HIS3 selective marker gene.

### Co-IP verification of Y2H interacting pairs

To test interactions identified in the AD-wrmcDNA library Y2H screen using co-IP (Figure 3, Additional file 5: Supplemental Table S10), the full length ORF coding for the target protein identified was amplified from the AD-wrmcDNA library, Gateway cloned into the pDONR201 Entry vector and transferred using the LR reaction into the expression vector pDEST-CMV-MYC which contains a MYC tag upstream of the B1 recombination site. For each fragment the endogenous Stop codon was preserved before the B2 recombinational tail (the endogenous C-terminus of the corresponding protein fragment was preserved).

To test interactions identified in the AD-wrmcDNA or AD-ORFeome screens, or both (see Additional file 6, Additional file 5: Supplemental Table S11), but using a B2-tailed construct for each PDZ-domain interacting protein, clones corresponding to the full length protein in the pDONR201 entry vector were retrieved from the *C. elegans *ORFeome collection [19]. Because of the nature of the constructs used in the ORFeome, LR-transfer of the ORF into the pDEST-CMV-MYC expression vector produced a protein ending with the C-terminal amino-acid sequence PAFLYKVVIIHSSMHLEGPIL (B2 encoding tail + 13 aa on pDEST-CMV-MYC before Stop codon).

Internal interactions (Figure 4, Additional file 7, Additional file 5: Supplemental Table S12 and S13) were tested by co-IP using 74 interactions for which the pair of PDZ/interacting proteins was found multiple times through the screening process of the AD-wrmcDNA library but that had no C-terminal consensus PDZ binding motif. These interactions corresponded to 59 different interacting proteins. To ensure a maximum reproducibility with the Y2H interactions, sequence data from the Y2H screen was used to define the smallest cDNA fragment identified among all clones obtained for each interaction in the Y2H screen (designated as the minimal interacting region, or MIR). For each fragment the codons encoding for the last three amino acid were removed from the primers and replaced by a Stop codon, giving rise after PCR amplification, Gateway cloning into the pDONR201 Entry vector and LR-transfer into the pDEST-CMV-MYC expression vector, to a cloned fragment encoding a protein lacking the last three amino acids. This was done to ensure that proteins could not interact by their native C-terminus, so that a positive result would provide support for an internal mode of interaction. When a PDZ-interacting protein was present in multiple pairs of interactions the smallest cDNA fragment of all pairs was used to test all interactions.

For all Co-IP experiments in this study, DNA encoding each PDZ domains tested was transferred from the pDONR201 Entry vector to the pDEST-CMV-3xHA expression vector containing the 3 × HA sequence upstream of the B1 recombination site.

Plasmids pDEST-CMV-3xHA and pDEST-CMV-MYC expressing their fusion proteins from the CMV promoters were transfected into 293T cells using Fugen 6 transfection reagent according to the manufacturers instructions (Roche). Cells were cultured for 48 hours in DMEM medium, and lysed in 0.1% NP-40 buffer (50 mM Tris-HCl, pH 7.5, 150 mM NaCl, 1 mM EDTA and complete protease inhibitors and phosphatase inhibitors (Thermo Scientific)). Lysates were cleared by centrifugation at 14,000 × g and subjected to co-immunoprecipitations of protein complexes using anti-HA (clone12CA5) sepharose beads. Purified complexes and control lysate (10 μg of total protein) samples were separated on Nu-PAGE Bis-Tris 4-12% gels (Invitrogen), and MYC and HA tagged proteins were detected using standard immunoblotting techniques. Antibodies used were mouse monoclonal anti-MYC (clone 9E10, Sigma) and monoclonal anti-HA (clone HA.11, Covance).

### Database searches

The Textpresso database [52] was used to search for interactions were both bait and prey proteins had public alphanumeric gene names. GO terms attributes were retrieved from Wormbase. Note that in *C. elegans *most attributes are currently inferred from electronic annotation (IEA).

## Authors' contributions

NL, JP, SB and SO carried out the experiments. NL performed the bioinformatic analysis. NL, JP and JR analyzed the data. JP, JPB and JR conceived the study. JR wrote the manuscript. All authors have read and approved the final manuscript.

## Supplementary Material

Additional file 1 **Definition of consensus classes**. Additional file 1 is a table describing the consensus classes used in this study. We defined for this study three extended consensus classes encompassing the different definitions available so far, so as to have the broadest definition of classes [1,10]. Click here for file

Additional file 2 **PDZ domains cloning and annotations**. Additional file 2 contains two tables (S1 and S2) listing for each PDZ domain identified, respectively the primer sequences and the Gene Ontology annotations. Supplemental Table S1: List of primers used to clone PDZ domains. Proteins names and IDs are given according to Wormbase WS150 [44]. For multiple PDZ domains in the same protein, a ".n" extension was added to the ID of the PDZ containing protein. This extension was numbered from the ATG (eg: F54E7.3.1 is the ID for the first PDZ domain of F54E7.3). When only one PDZ was present, protein ID was kept as such. Coordinates on PDZ domain containing proteins correspond to the splice form specified in the third column (PDZ domain containing protein ID). Each primer contains the B1 and B2 Gateway recombination cloning tail. Supplemental Table S2: Gene Ontology annotation based on experimental data for PDZ domains proteins. Gene ontology annotations were retrieved from Wormbase WS190 [16]. Experimental Evidence Codes: EXP: Inferred from Experiment, IDA: Inferred from Direct Assay, IPI: Inferred from Physical Interaction, IMP: Inferred from Mutant Phenotype, IGI: Inferred from Genetic Interaction, IEP: Inferred from Expression Pattern. Click here for file

Additional file 3 **PDZome network**. Additional file 3 contains four tables (S3 to S6) listing the PDZome network interacting pairs and giving statistical analysis of the interactions. Supplemental Table S3: Two hybrid screen results. Gene names and ID are given according to Wormbase WS150[44]. Number of hits refers to the number of independent colonies identified and phenotypically tested for each interacting partner. Number of splice-forms identified or predicted in Wormbase WS150 and last 6 amino acids of each splice-form are given in cases were several splice-forms are identified or predicted. When sequencing from the N-terminus did not span the entire fragment, and thus the C-terminus was not experimentally confirmed, if any of the predicted splice-form had a C-terminal consensus motif, to be conservative, a consensus class was attributed. Consensus class type: [ST]X[YFWCMVILA] = 1; [YFWCMVILA]X[YFWCMVILA] = 2; [DE]X[YFWCMVILA] = 3 (X: any amino acid). Supplemental Table S4: Promiscuity and specificity of PDZ interactome network. Number of independent interacting proteins per PDZ domain, and number of PDZ domains interacting with each protein are given for AD-wrmcDNA and AD-ORFeome libraries two-hybrid screens. Supplemental Table S5: Promiscuity and specificity of PDZ interactome network: mean and median for number of interacting proteins per PDZ or vice versa. Supplemental Table S6: Number of interacting proteins per consensus class in network. Single hits: interacting proteins for which only one clone was identified in two-hybrid screens. Multiple hits: interacting proteins for which more than one clone was identified in two-hybrid screens. Click here for file

Additional file 4 **Functional annotation of interacting proteins**. Additional file 4 contains three tables (S7 to S9) listing the functional annotations of the PDZ domain interacting proteins. Supplemental Table S7: Concise description, GO terms and KOG (EuKaryotic Orthologous Groups), for each interacting protein when available, retrieved from Wormbase WS 190 [16]; note that most attributes are inferred from electronic annotation. Experimental Evidence Codes: EXP: Inferred from Experiment, IDA: Inferred from Direct Assay, IPI: Inferred from Physical Interaction, IMP: Inferred from Mutant Phenotype, IGI: Inferred from Genetic Interaction, IEP: Inferred from Expression Pattern. Computational Analysis Evidence Codes: ISS: Inferred from Sequence or Structural Similarity, ISO: Inferred from Sequence Orthology, ISA: Inferred from Sequence Alignment, ISM: Inferred from Sequence Model, IGC: Inferred from Genomic Context, RCA: inferred from Reviewed Computational Analysis. Author Statement Evidence Codes: TAS: Traceable Author Statement, NAS: Non-traceable Author Statement. Curator Statement Evidence Codes: IC: Inferred by Curator, ND: No biological Data available. Automatically-assigned Evidence Codes: IEA: Inferred from Electronic Annotation. Supplemental Table S8: Classification of interacting proteins according to Cellular Components terms: integral to membrane, nucleus and other&unknown. Supplemental Table S9: Classification of interacting proteins according to Biological Processes: manual curation of annotations retrieved from Wormbase WS190 were used to define the 11 groups of processes shown in Figure 2. Click here for file

Additional file 5 **Verification of two-hybrid interacting pairs by co-immunoprecipitation**. Additional file 5 contains four tables (S10 to S13) listing interacting proteins pairs tested and results. Supplemental Table S10 lists tested pairs involving PDZ domains and their respective two-hybrid identified interacting proteins possessing a free C-terminus. Supplemental Table S11 lists tested pairs involving PDZ domains and their respective two-hybrid identified interacting proteins using a B2 tailed construct. Supplemental Table S12 lists the 59 protein fragments that did not possess a C-terminal binding motif tested by co-immunoprecipitation in a C-terminally truncated form against their respective two hybrid interacting PDZs. Sequences where there was creation of a new C-terminal consensus sites after truncation are shown. Supplemental Table S13 lists the primers pairs used to amplify and clone truncated interacting protein fragments used in the co-immunoprecipitation experiment. Click here for file

Additional file 6 **Co-IP verification of yeast two hybrid interacting pairs identified in AD-wrmcDNA and AD-ORFeome screens using a B2 tailed construct**. Additional file 6 is a figure showing the Co-IP verification of yeast two hybrid interacting pairs identified in AD-wrmcDNA and AD-ORFeome screens using a B2 tailed construct. (A) Schematic representation of PDZ domains carrying N-terminal 3XHA epitope tag and of their interacting protein, ending with the B2 tail, carrying N-terminal MYC epitope. (B) Each pair of constructs to be tested was co-expressed in 293T cells and co-IP was performed using cellular lysates subjected to precipitation with anti-HA sepharose. Presence of interacting protein upon precipitation was revealed by western blotting using anti-MYC serum. For each IP performed three panels are presented. Upper panel: IP reaction probed upon resolution on SDS-PAGE and blotting with anti-HA antibody detecting HA-PDZ domain; Middle panel: the same IP reaction probed with anti-MYC serum detecting ORF (MYC:ORF); lower panel: detection of expression of each ORF by probing total crude cellular extracts (input) with anti-MYC serum. Table summarizes the interaction pairs tested and color code is used to indicate the outcome (purple: interaction tested positive, grey: no interaction and yellow: inconclusive as one or both partners are not expressed). (C) Each ORF used in above co-IP experiment was also subjected to co-transfection and co-immunoprecipitation with empty pDEST-CMV-3xHA vector to serve as a negative control for the binding assay. Detection and analysis were performed as above. Click here for file

Additional file 7 **Negative controls of immunoprecipitations shown in Figure **4. Additional file 7 is a figure showing the test for unspecific binding of non-consensus C-terminally truncated proteins (MYC:MIRdCter) to irrelevant HA epitoped peptide in co-immunoprecipitation reaction corresponding to negative control of the experiment described in Figure 4. Each MYC:MIRdCter construct was co-expressed in 293T cells together with empty pDEST-CMV-3xHA vector and co-IPed using anti-HA sepharose beads. Binding of given protein upon precipitation was revealed by western blotting using anti-MYC serum. For each IP performed three panels are presented. Upper panel: IP reaction probed after resolution on SDS-PAGE and blotting with anti-HA antibody. Middle panel: the same IP reaction probed with anti-MYC serum detecting the truncated protein fragments (MYC:MIRdCter). Lower panel: detection of expression of each truncated protein fragment by probing total crude cellular extracts (input) with anti-MYC serum. Click here for file
