# Supplementary material for: A genome-wide study of PDZ-domain interactions in C. elegans reveals a high frequency of non-canonical binding
Source: BMC Genomics. 2010 Nov 26;11:671. doi: 10.1186/1471-2164-11-671 (PMC3091786; doi:10.1186/1471-2164-11-671)
Supplement: Additional file 1 — Definition of consensus classes. Additional file 1 is a table describing the consensus classes used in this study. We defined for this study three extended consensus classes encompassing the different definitions available so far, so as to have the broadest definition of classes [1,10]. [file 1471-2164-11-671-S1.PDF]

	Extended consensus used to define Classes in this study
Class 1	[ST]X[FWCYMVILA]
Class 2	[YFWCMVILA]X[YFWCMVILA]
Class 3	[DE]X[YFWCMVILA]
	X any amino acid

	Nourry et al. 2003
Class 1	[ST]X[VLI]
Class 2	[φ]X[φ]
Class 3	[DE]X[φ]
	φ hydrophobic (V,I,L,F,Y)
	X any amino acid

	Tonikian et al. 2008
Class 1a	φ[K/R]XSDV
Class 1b	Ω[R/K]ET[S/T/R/K]φ
Class 1c	φφETXL
Class 1d	ETXV
Class 1e	TWΨ
Class 1f	ΩΩTWΨ
Class 1g	φφφ[T/S][T/S]ΩΨ
Class 1h	φφ[D/E][T/S]WΨ
Class 2a	FDΩΩC
Class 2b	WXΩFDV
Class 2c	WΩφDΨ
Class 2d	φφX[E/D]φφφ
Class 2e	φφφφ
Class 2f	[D/E]φΩφ
Class 3a	WΩ[S/T]DWΨ
Class 4a	ΩφGWF
	φ hydrophobic (V,I,L,F,W,Y,M)
	Ω aromatic (F, W,Y);
	Ψ aliphatic (V, I, L, M);
	X any amino acid
