# Supplementary material for: A genome-wide study of PDZ-domain interactions in C. elegans reveals a high frequency of non-canonical binding
Source: BMC Genomics. 2010 Nov 26;11:671. doi: 10.1186/1471-2164-11-671 (PMC3091786; doi:10.1186/1471-2164-11-671)
Supplement: Additional file 6 — Co-IP verification of yeast two hybrid interacting pairs identified in AD-wrmcDNA and AD-ORFeome screens using a B2 tailed construct. Additional file 6 is a figure showing the Co-IP verification of yeast two hybrid interacting pairs identified in AD-wrmcDNA and AD-ORFeome screens using a B2 tailed construct. (A) Schematic representation of PDZ domains carrying N-terminal 3XHA epitope tag and of their interacting protein, ending with the B2 tail, carrying N-terminal MYC epitope. (B) Each pair of constructs to be tested was co-expressed in 293T cells and co-IP was performed using cellular lysates subjected to precipitation with anti-HA sepharose. Presence of interacting protein upon precipitation was revealed by western blotting using anti-MYC serum. For each IP performed three panels are presented. Upper panel: IP reaction probed upon resolution on SDS-PAGE and blotting with anti-HA antibody detecting HA-PDZ domain; Middle panel: the same IP reaction probed with anti-MYC serum detecting ORF (MYC:ORF); lower panel: detection of expression of each ORF by probing total crude cellular extracts (input) with anti-MYC serum. Table summarizes the interaction pairs tested and color code is used to indicate the outcome (purple: interaction tested positive, grey: no interaction and yellow: inconclusive as one or both partners are not expressed). (C) Each ORF used in above co-IP experiment was also subjected to co-transfection and co-immunoprecipitation with empty pDEST-CMV-3xHA vector to serve as a negative control for the binding assay. Detection and analysis were performed as above. [file 1471-2164-11-671-S6.PDF]

A

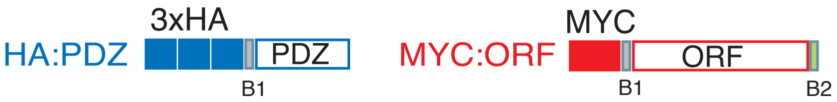

B

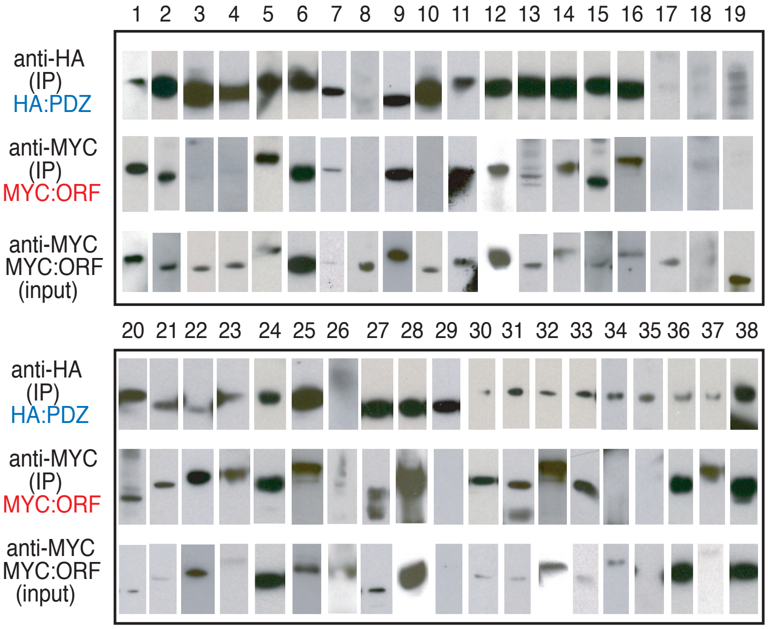

interaction confirmed

interaction not confirmed

lack of protein expression

sample no.	HA:PDZ	MYC:ORF	sample no.	HA:PDZ	MYC:ORF
1	C01B7.4	Y105E8A.6	20	F54E7.3.1	T11B7.1
2	C01B7.5	Y55F3BR.6	21	F54E7.3.2	F53A10.2
3	C01B7.5	B0547.1	22	K01A6.2.1	C34C6.6
4	C01F6.6.1	B0547.1	23	K01A6.2.2	F54D10.7
5	C09H6.2.1	F32A6.4	24	K01A6.2.3	Y48E1B.12
6	C09H6.2.2	Y48E1B.12	25	K01A6.2.4	Y77E11A.7
7	C25F6.2.1	F53A10.2	26	R11A8.8.2	F32A6.4
8	C25F6.2.1	B0547.1	27	T05C12.6	F53F10.1
9	C25F6.2.3	C34C6.6	28	T05C12.6	Y39A1A.3
10	C27A2.6	B0547.1	29	T05C12.6	C28H8.12
11	C33B4.3	H06I04.1	30	T26E3.3	Y105E8A.6
12	C34F11.9	Y39A1A.3	31	T26E3.3	F53B3.1
13	C34F11.9	Y40C5A.1	32	T26E3.3	Y77E11A.7
14	C34F11.9	F54D10.7	33	T26E3.3	H06I04.1
15	C34F11.9	Y55F3BR.6	34	T26E3.3	C28H8.12
16	C34F11.9	Y77E11A.7	35	T26E3.3	W03G1.3
17	C34F11.9	B0547.1	36	W03F11.6	Y48E1B.12
18	C44B7.1	F37A4.9	37	W03F11.6	F54D10.7
19	C52A11.4.2	K06A4.5	38	Y54G11A.10	Y48E1B.12

C

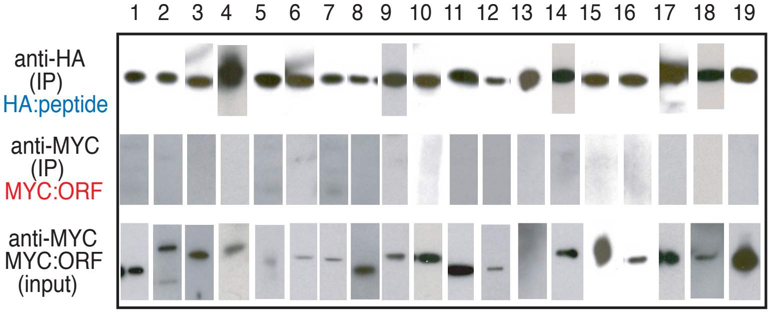

sample no.	MYC:ORF	sample no.	MYC:ORF
1	B0547.1	11	K06A4.5
2	C28H8.12	12	T11B7.1
3	C34C6.6	13	W03G1.3
4	F32A6.4	14	Y105E8A.6
5	F37A4.9	15	Y39A1A.3
6	F53A10.2	16	Y40C5A.1
7	F53B3.1	17	Y48E1B.12
8	F53F10.1	18	Y55F3BR.6
9	F54D10.7	19	Y77E11A.7
10	H06I04.1		
