# Supplementary material for: A genome-wide study of PDZ-domain interactions in C. elegans reveals a high frequency of non-canonical binding
Source: BMC Genomics. 2010 Nov 26;11:671. doi: 10.1186/1471-2164-11-671 (PMC3091786; doi:10.1186/1471-2164-11-671)
Supplement: Additional file 7 — Negative controls of immunoprecipitations shown in Figure 4. Additional file 7 is a figure showing the test for unspecific binding of non-consensus C-terminally truncated proteins (MYC:MIRdCter) to irrelevant HA epitoped peptide in co-immunoprecipitation reaction corresponding to negative control of the experiment described in Figure 4. Each MYC:MIRdCter construct was co-expressed in 293T cells together with empty pDEST-CMV-3xHA vector and co-IPed using anti-HA sepharose beads. Binding of given protein upon precipitation was revealed by western blotting using anti-MYC serum. For each IP performed three panels are presented. Upper panel: IP reaction probed after resolution on SDS-PAGE and blotting with anti-HA antibody. Middle panel: the same IP reaction probed with anti-MYC serum detecting the truncated protein fragments (MYC:MIRdCter). Lower panel: detection of expression of each truncated protein fragment by probing total crude cellular extracts (input) with anti-MYC serum. [file 1471-2164-11-671-S7.PDF]

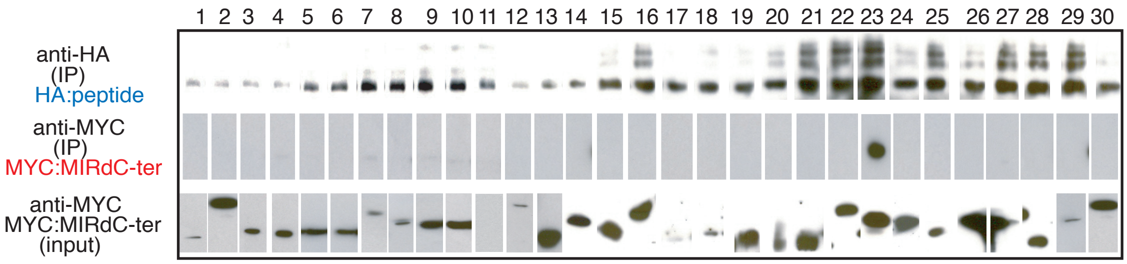

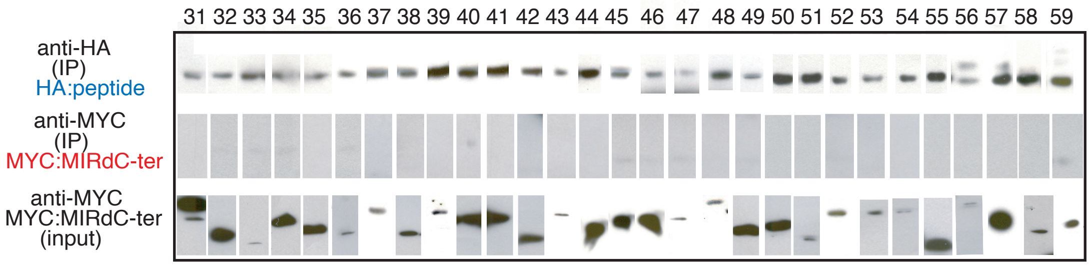

sample no.	MYC:MIRdC-ter
**1**	B0001.6
**2**	B0365.1
**3**	C03A7.14
**4**	C03A7.4
**5**	C03A7.8
**6**	C04C3.3
**7**	C07E3.6
**8**	C09B8.6
**9**	C17E7.4
**10**	C18A11.7
**11**	C27A12.7
**12**	C37A5.9
**13**	C39D10.7
**14**	C50B6.2
**15**	C56G2.7
**16**	F07A11.2
**17**	F09E5.7
**18**	F12F6.1
**19**	F17E9.5
**20**	F20D1.1
**21**	F23F1.8
**22**	F32G8.6
**23**	F33G12.5
**24**	F39G3.3
**25**	F43D9.4
**26**	F52E4.6
**27**	F53B3.1
**28**	F53F10.1
**29**	F54B11.5
**30**	K04D7.1

sample no.	MYC:MIRdC-ter
**31**	K08E3.5
**32**	K08F8.4
**33**	K12F2.1
**34**	K12H4.7
**35**	R04E5.10
**36**	R06F6.12
**37**	R09B5.5
**38**	R148.3
**39**	T09E8.1
**40**	T19B4.5
**41**	T20H4.3
**42**	W03D8.9
**43**	W04D2.1
**44**	W10D9.4
**45**	Y105E8A.6
**46**	Y37E11AL.3
**47**	Y37E3.11
**48**	Y38C1BA.2
**49**	Y40C5A.1
**50**	Y51A2D.15
**51**	Y54E2A.11
**52**	Y55F3BR.6
**53**	Y56A3A.32
**54**	Y59A8B.22
**55**	Y71F9B.3
**56**	Y71G12B.9
**57**	Y77E11A.7
**58**	ZK1067.7
**59**	ZK836.1
